# Doxorubicin/Nucleophosmin Binding Protein-Conjugated Nanoparticle Enhances Anti-leukemia Activity in Acute Lymphoblastic Leukemia Cells *in vitro* and *in vivo*

**DOI:** 10.3389/fphar.2021.607755

**Published:** 2021-05-28

**Authors:** Donghui Gan, Yuwen Chen, Zhengjun Wu, Liping Luo, Shimuye Kalayu Yirga, Na Zhang, Fu Ye, Haijun Chen, Jianda Hu, Yingyu Chen

**Affiliations:** ^1^ Department of Hematology, Fujian Institute of Hematology, Fujian Provincial Key Laboratory of Hematology, Fujian Medical University Union Hospital, Fuzhou, China; ^2^ College of Chemistry, Fuzhou University, Fuzhou, China

**Keywords:** acute lymphoblastic leukemia, p53, apoptosis, doxorubicin (dox), multidrug resistance (MDR), nanoparticle, nucleophosmin (NPM)

## Abstract

Acute lymphoblastic leukemia (ALL) is an aggressive malignancy. Adults with ALL have more than 50% relapse rates. We have previously validated that overexpression of nucleophosmin (NPM) is involved in the multidrug resistance (MDR) development during ALL; and a synthetically engineered recombinant NPM binding protein (NPMBP) has been developed in our group; NPMBP and doxorubicin (DOX) can be conjugated in a nanoparticle-based drug delivery system named DOX-PMs-NPMBP to counteract MDR during ALL. Here, we evaluated the antileukemia potential of DOX-PMs-NPMBP in resistant ALL cells. This study demonstrates that DOX-PMs-NPMBP significantly enhances chemosensitivity to DOX in ALL cells. Despite at variable concentrations, both resistant and primary ALL cells from relapsed patients were sensitive to DOX-PMs-NPMBP. In detail, the half maximal inhibitory concentration (IC50) values of DOX-PMs-NPMBP were between 1.6- and 7.0-fold lower than those of DOX in cell lines and primary ALL cells, respectively; and apoptotic cells ratio was over 2-fold higher in DOX-PMs-NPMBP than DOX. Mechanistically, p53-driven apoptosis induction and cell cycle arrest played essential role in DOX-PMs-NPMBP-induced anti-leukemia effects. Moreover, DOX-PMs-NPMBP significantly inhibited tumor growth and prolonged mouse survival of ALL xenograft models; and no systemic toxicity occurrence was observed after treatment during follow-up. In conclusion, these data indicate that DOX-PMs-NPMBP may significantly exert growth inhibition and apoptosis induction, and markedly improve DOX antileukemia activity in resistant ALL cells. This novel drug delivery system may be valuable to develop as a new therapeutic strategy against multidrug resistant ALL.

## Introduction

Acute lymphoblastic leukemia (ALL) is an aggressive, malignant disease. Improvements in multiagent chemotherapy treatments, along with tailored risk assessment, have raised survival rates in pediatric ALL. However, adult ALL patients still have a relapse rate exceeding 50%, and an overall survival rate of 20–40% (Sive et al., 2012; Tasian and Gardner, 2015; Richard-Carpentier et al., 2019). In recent years, a personalized treatment approach involving the following has contributed to the progress of adult ALL therapy: response to minimal residual disease and disease genetics; adopting pediatric-inspired regimens in younger adults; advances in transplantation; incorporation of new treatments and tyrosine kinase inhibitors found in frontline regimens; as well as adoptive cellular therapy (Aldoss and Stein, 2018; Pillai et al., 2019). In particular, antibody-based therapies, such as monoclonal antibodies and antibody-drug conjugates have shown promising activity profiles for the treatment of ALL (Phelan and Advani, 2018; Schmied et al., 2019). Introduction of these strategies in the frontline setting has been ongoing and will likely unravel significant benefits for ALL.

Nanomedicines is a unique treatment strategy that offers different methods of administering drugs and improvement of the therapeutic efficacy and poses less side effects to healthy cells and tissues. In the context of drug resistance, nanoparticles have been shown to improve P-glycoprotein (P-gp) efficacy, suggesting that nanomedicine might overcome multidrug resistance (MDR) (Dong and Mumper, 2010; Mattheolabakis et al., 2012). Nucleophosmin (NPM) is a multifunctional nucleolar protein involved in several biological processes, as well as the pathogenesis of malignancies in humans (Borer et al., 1989; Colombo et al., 2011). NPM mutations induce the nuclear export of the mutants, while aberrant cytoplasmic delocalization of NPM is of high importance for leukaemogenesis (Liso et al., 2008; Chen and Hu, 2020). We have previously validated that overexpression of NPM and nucleolin (NCL) is involved in the MDR development and an important indicator for prognosis evaluation in ALL (Hu et al., 2011). Additional research demonstrated that RNA interference causes a knockdown of NPM, which can reverse MDR in resistant leukemic cells (Lin et al., 2013; Wang et al., 2015), and a synthetically engineered recombinant NPM binding protein (NPMBP) has been developed. Furthermore, NPMBP and doxorubicin (DOX) were bound to nanoparticle-based drug delivery system named DOX-PMs-NPMBP (China patent application 2020103023956, 2020107395750), in the attempt to counteract MDR during ALL, but this requires pharmacological proof. Here, we evaluated the anti-leukemia potential of DOX-PMs-NPMBP for ALL. We report that such strategy can markedly improve the antileukemia effect of DOX, exposing a new therapeutic strategy for ALL, particularly multidrug resistant ALL.

## Materials and Methods

### Cell Culture and the Induction of DOX-Resistant Nalm6 Cells

Nalm6 cells were obtained from Fujian Medical University (Fuzhou, China). The drug-resistance induction was as previous described (Wang et al., 2015). Briefly, Nalm6 cells were exposed to incrementally higher levels of DOX to establish a leukemia cell line that was resistant to DOX (Hanhui Pharmaceuticals Co., LTD., Shanghai, China). The drug sensitivity between the resistant cells and the parent cells was compared with a 3-(4,5-dimethylthiazol-2-yl)-2,5-diphenyltetrazolium bromide (MTT, Sigma, St. Louis, MO, United States) assay. Prior to each experiment, cells were grown in a culture medium free of DOX for a minimum of two weeks.

Either Nalm6 cells or Nalm6/DOX cells were further transduced with a lentiviral vector that encoded the luciferase (Luc) gene. The efficiency of the stable transduction was monitored by fluorescence microscopy. Both cell lines with stable luciferase expression were named as Nalm6-Luc cells and Nalm6-Luc/DOX cells, respectively.

### Primary Cell Isolation from Peripheral Blood

Peripheral blood (PB) samples were isolated from six ALL patients at our institute. All patients were diagnosed with ALL according to World Health Organization (WHO) and standard French-American-British (FAB) standards. All patients provided their informed consent according to the Helsinki declaration, while the institutional review board of the Ethics Committee of Fujian Medical University Union Hospital approved the study (2018–113). Primary ALL cells were isolated from PB samples as previously reported (Chen et al., 2014; Chen et al., 2020).

### Cloning, Protein Expression and Purification

**FIGURE 1:**
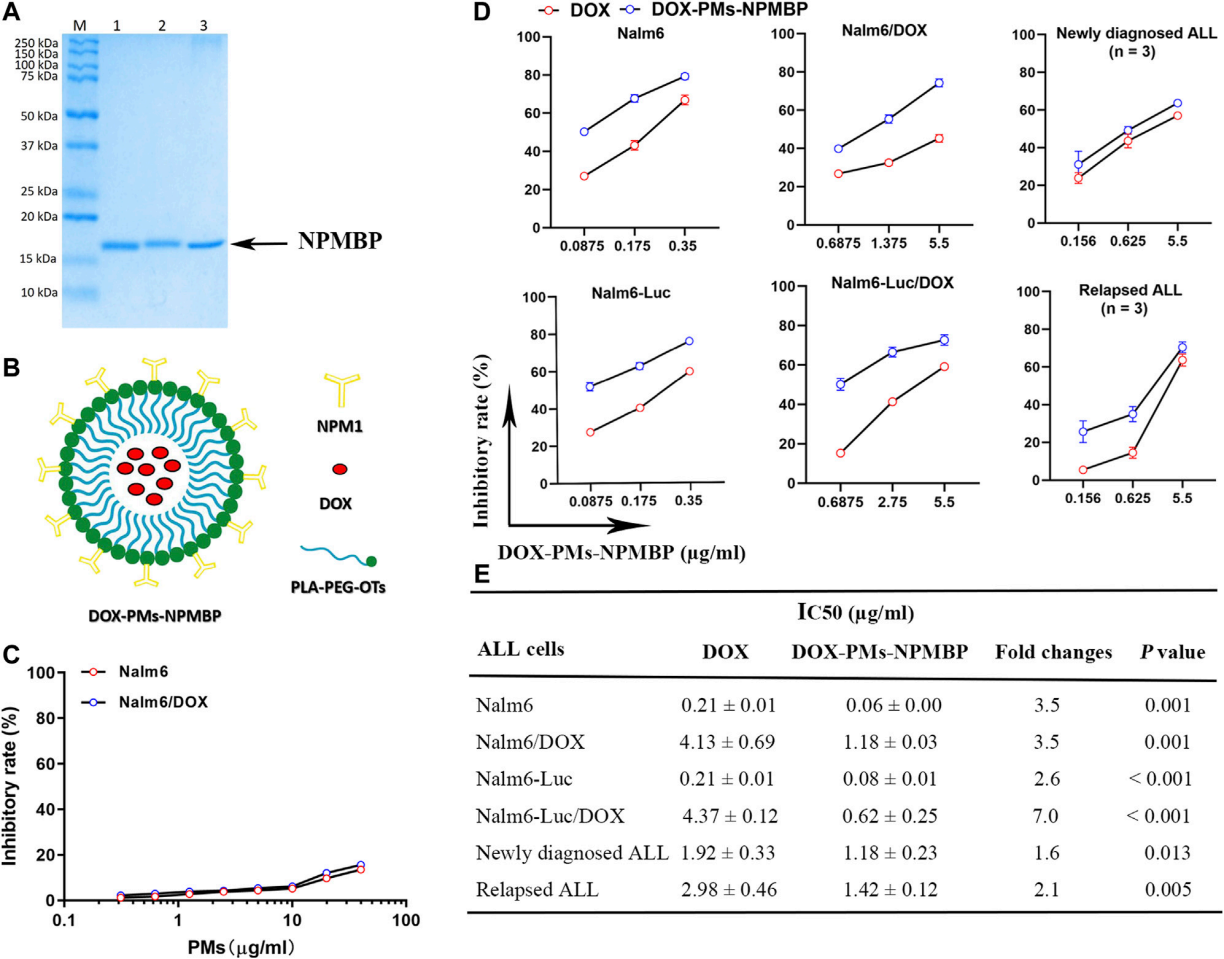
IC50 values of DOX-PMs-NPMBP on multiple ALL cell lines and different primary ALL cells from patients as measured by MTT assay. **(A)** Representative gel demonstrating the expected molecular weight and purity of NPMBP. **(B)** Schematic illustration of the structure of DOX-PMs-NPMBP. **(C)** Quantitative evaluation of inhibitory rates in Nalm6-Luc cells and Nalm6-Luc/DOX cells with equivalent concentrations of polymeric micelles (PMs) for 48 h. **(D)** Quantitative evaluation of inhibitory rate in various ALL cells with DOX or DOX-PMs-NPMBP indicated concentration. **(E)** ALL cell lines or primary cells from six patients treated with increasing concentrations of DOX or DOX-PMs-NPMBP for 48 h. IC50 values of DOX and DOX-PMs-NPMBP were calculated. Data are presented as mean ± SD of three independent experiments.

NPMBP was produced in accordance with the standard procedure by Wuhan Institute of Biotechnology (Wuhan, China). Briefly, NPM truncation constructs were cloned in a pET28a vector, and recombinant his-tagged NPMBP was expressed in *E. coli* BL21 strain in medium with the presence of 50 mg/l Kanamycin. Bacterial cultures wore grown at 37°C to an optical density OD600 0.5–0.8. Protein expression was induced by the addition of 0.8 mM Isopropyl β-D-Thiogalactoside (IPTG, GoldBio, st. Louis, United States), the temperature was lowered to 25°C and the cultures were incubated for 12 h. Bacterial cultures were harvested by centrifugation and lysed by sonication on ice. NPMBP with high binding affinity was screened and isolated by phage display technology, which was further purified using Ni (2+) affinity chromatographic column. SDS-PAGE showed a highly pure 16 kD protein, in accordance with the expected molecular weight (Figure 1A). The peptide with best binding affinity towards NPM was identified (Supplementary Figure 1). Purified proteins were further concentrated and reconstituted in phosphate-buffered saline (PBS) containing 5% glycerol, pH 7.4 at −80°C.

### Preparation of DOX-PMs-NPMBP

Polyethylene glycol (PEG) was employed to modify the hydrophobic poly-(lactic acid) (PLA) to form the polymeric micelles (PMs) according to previous reports (Aliabadi et al., 2007; Duncan et al., 2019; Hoang et al., 2019). Equal concentrations of DOX and NPMBP at 0.3 mg/ml, achieving high drug-loading capacity and the high colloidal stability, were conjugated polymer-based nanoparticles and micelles. This drug- and binding protein-coated nanoparticle was named as DOX-PMs-NPMBP (Figure 1B, China Patent Application 2020103023956, 2020107395750). The DOX-PMs-NPMBP stock solutions in PBS at pH 7.4 were stored at −20°C.

### Cell Viability Assay

ALL cells (4.0 × 10^5^ cells/ml for cell lines and 2.5 × 10^6^ cells/ml for primary cells) were seeded in 96-well plate. Cells were incubated directly after plating in triplicate with serial concentrations of DOX-PMs-NPMBP, DOX, and PMs. Cell viability was determined by MTT assay as we previously reported (Chen et al., 2020). The half maximal inhibitory concentration (IC50) was determined by the Logit way.

### Cell Apoptosis and Cell Cycle Distribution Assessed by Flow Cytometry

A total of 3.0 × 10^5^ Nalm6/DOX cells were seeded in 6-well plate with 3 ml per well of medium. Cells were then treated with DOX, DOX-PMs-NPMBP and vehicle control for 24 h. The staining process of harvested cell pellets was manipulated using Annexin V Apoptosis Detection Kit (Becton Dickinson, CA, United States) according to the manufacturer’s protocols. Frequency of apoptotic cells was analyzed by flow cytometry (BD FACSVerse^TM^, Becton Dickinson, CA, United States). For cell cycle analysis, a total 3.0 × 10^6^ cells were treated with DOX or DOX-PMs-NPMBP for 24 h. Cell cycle distribution was examined as previously described (Chen et al., 2020). Propidium iodide used in this assay was purchased from BD Biosciences.

### Cellular Uptake of DOX

A total of 4.0 × 10^5^ Nalm6/DOX cells per well were plated in 6-well plates in 2 ml of growth medium. Cells were then incubated with or without DOX, DOX-PMs-NPMBP at 37°C for 12 h, cells were twice washed with ice-cold PBS. Flow cytometer analysis was used to assess the cell-associated mean fluorescence intensity (MFI) of the DOX with excitation/emission wave lengths of 485/580 nm.

### Rhodamine123 (Rho123) Efflux Assay

Nalm6/DOX cells were seeded and treated with or without DOX, DOX-PMs-NPMBP as the procedures for the measurement of cellular uptake of DOX. Then, for the drug efflux ability analysis, Rho123 at 2 μM was added to each well and incubated for another 30 min at 37°C. After that, cells were twice washed with ice-cold PBS. Flow cytometer analysis was used to assess the MFI of Rho123 in the Nalm6/DOX cells through FITC channel.

### RNA Extraction and Sequencing

Nalm6/DOX cells were grown in the presence or absence of DOX or DOX-PMs-NPMBP for 12 h. After treatment, cells were pelleted by centrifugation and total RNA was extracted using TRIzol reagent (Invitrogen, CA) and quantified by UV spectrophotometry (Nanodrop). For each sample 1 µg RNA was inputted when preparing the RNA samples. A NEBNext® UltraTM RNA Library Prep Kit for Illumina® (NEB, United States) was used to create the sequencing libraries according to the manufacturer’s instructions. We added index codes to designate sequences for each sample. A cBot Cluster Generation System with TruSeq PE Cluster Kit v3-cBot-HS (Illumina) was used to cluster the index-coded samples according to the manufacturer’s instructions. After the clusters were generated, an Illumina Novaseq platform was used to sequence the library and 150 bp paired-end reads were created. The RNA sequencing and the bioinformatic analysis were done by Novogene Co., Ltd. (Beijing, China).

### Quantitative Real-Time Polymerase Chain Reaction

Quantitative real-time polymerase chain reaction (qRT-PCR) was performed on the ABI prism 7,700 sequence detection system (Applied Biosystems, CA, United States) using EvaGreen MasterMix-Low ROX kit (Richmond, BC, Canada). Table 1 lists primer sequences for glyceraldehyde-3-phosphate dehydrogenase (GAPDH), NPM, proto-oncogene c-myc, TP53 (p53) and p14ARF, B-cell lymphoma 2 (bcl-2), p21^WAF1/CIP1^, bcl-2 associated X (bax), mouse double minute 4 (MDM4), MDR, and phosphatase and tensin homolog (PTEN) detection by qRT-PCR. The relative mRNA amounts were calculated by the 2^−∆∆Ct^ method as we previously reported (Chen et al., 2020).

**TABLE 1:** The primer sequences for qRT‐PCR

Gene	Sequence
GAPDH	F: 5′‐CCA​CCA​TGG​AGA​AGG​CTG​GGG​CTC​A-3′
	R: 5′‐ATC​ACG​CCA​CAG​TTT​CCC​GGA​GGG​G-3′
NPM	F: 5′‐GTA​CAG​CCA​ACG​GTT​TCC​CTT​G-3′
	R: 5′‐TTC​ACA​TCC​TCC​TCC​TCT​TCA​TCT​TC-3′
c-myc	F: 5′‐TCC​TGG​CAA​AAG​GTC​AGA​GT-3′
	R: 5′‐TCT​GAC​ACT​GTC​CAA​CTT​GAC-3
p53	F: 5′‐CAG​CAC​ATG​ACG​GAG​GTT​GT-3′
	R: 5′‐TCA​TCC​AAA​TAC​TCC​ACA​CGC-3′
bcl-2	F: 5′‐ACG​ACT​TCT​CCC​GCC​GCT​AC-3′
	R: 5′‐CTG​AAG​AGC​TCC​TCC​ACC​AC-3′
p21	F: 5′‐CGA​TGG​AAC​TTC​GAC​TTT​GTC​A-3′
	R: 5′‐GCA​CAA​GGG​TAC​AAG​ACA​GTG-3′
bax	F: 5′‐ATG​GAG​CTG​CAG​AGG​ATG​ATT​G-3′
	R: 5′‐AAT​GTC​CAG​CCC​ATG​ATG​GTT​C-3′
p14ARF	F: 5′‐TCG​CGA​TGT​CGC​ACG​GTA-3′
	R: 5′‐CAA​TCG​GGG​ATG​TCT​GAG​GGA​C-3′
MDM4	F: 5′‐TGG​ACA​AAT​CAA​TCA​GGT​ACG​A-3′
	R: 5′‐CTC​CTG​CTG​ATC​ATA​AAG​TTG​C-3′
MDR	F: 5′‐CCC​ATC​ATT​GCA​ATA​GCA​GG-3′
	R: 5′‐GTT​CAA​ACT​TCT​GCT​CCT​GA-3′
PTEN	F: 5′‐TGC​AGT​ATA​GAG​CGT​GCA​GA-3′
	R: 5′‐TAG​CCT​CTG​GAT​TTG​ACG​GC-3′

### Western Blotting

Cells were cultured with or without DOX or DOX-PMs-NPMBP for 24 h. Western blot analysis was performed according to we previously described (Chen et al., 2014; Chen et al., 2020). Primary antibodies against Tublin, Actin and c-Myc were obtained from Santa Cruz Biotechnology, Inc. (Santa Gruz, CA, United States). NPM, P-gp, PTEN, Bcl-2, Bax, phospho-14ARF (p14ARF), p21^WAF1/CIP1^, PI3 Kinase p85, phospho-p85 (p-p85), p53 and phospho-p53 (p-p53), Cyclin D, Cyclin E, phospho-retinoblastoma (p-Rb), mouse double minute 2 (MDM2) and MDM4 antibodies were provided by Cell Signaling Technology (Danvers, MA, United States). GAPDH antibody was purchased from Abcam (Cambridge, United Kingdom).

### *In vivo* Anti-leukemic Effects in Nalm6-Luc/DOX Xenograft Nude Mice

Around 5 × 10^6^ Nalm6-Luc/DOX cells were subcutaneously transplanted to 6- to 8-week-old BALB/C-nude mice (Slac Laboratory Animal Co., Ltd. Shanghai, China). Twenty days after transplantation, the Nalm6-Luc/DOX xenograft models received either DOX, DOX-PMs-NPMBP, or PMs at maximal dose (equivalent to 3 mg/kg DOX) by intraperitoneal injection once daily for 3 days. PBS injection was administered as vehicle control. Animal body weight was monitored during the follow-up. Tumor size was measured during study course. Tumor inhibition rate were calculated as we previously reported (Chen et al., 2018). Nalm6-Luc/DOX xenograft models in response to treatment were monitored by IVIS LUMINA II Imaging system (Caliper Life Sciences, Hopkinton, MA, United States) at the 25th day after the initial treatment. The follow-up was completed at the 30th day, tumor tissues and organs were carefully excised and fixed in 10% formalin, embedded in paraffin. Then, tumor tissue sections were sliced, adhered to slide, and stained with hematoxylin-eosin (HE).

### Therapy of NCG Mouse Xenografted With Nalm6-Luc/DOX Cells

NOD-Prkdc^em26^Il2rg^em26^Nju (NCG) mice (Nanjing Biomedical Research Institute of Nanjing University, Nanjing, China) aged six-to eight-week-old were inoculated intravenously in their tail veins with 1.0 × 10^4^ Nalm6-Luc/DOX cells. Seven days later, recipient mice were intraperitoneally injected with either DOX or DOX-PMs-NPMBP at 1.5 mg/kg once daily for 3 days. Vehicle control mice were received PBS only. The mouse body weight was tracked and recorded. Either treated mice or vehicle control mice were monitored by IVIS LUMINA II Imaging System. Animals were sacrificed until the 14th day after start of the treatment. The liver and spleen were weighed. Bone marrow (BM) smears were obtained. Cell morphology was assessed microscopically upon Wright-Giemsa staining. BM cells were isolated and stained with anti-human CD19-APC monoclonal antibody (Becton Dickinson). CD19^+^ Nalm6-Luc/DOX in BM were analyzed by flow cytometry.

The experiment was repeated and all recipients were closely monitored. Survival times were recorded. Follow-up was completed on days 42 post leukemia cell inoculation. All animals were maintained at the animal facility in Fujian Medical University. Animal experiments were performed according to previously reported procedures (Chen et al., 2020). All protocols were reviewed and approved by the Ethics Committee of Institutional Animal Care and Use in Fujian Medical University (2017–071).

### Statistical Analysis

The data listed is that of the mean ± the standard deviation (SD). Statistical significance was determined using the two-tailed Student’s *t*-test or one-way analysis of variance (ANOVA). GraphPad Prism software (version 8.0) was used to conduct statistical analysis. Kaplan-Meier methods and a long-rank test were used to assess the life span of the mice. Statistically significant results were those *p* < 0.05, *p* < 0.01, *p* < 0.001, and *p* < 0.0001.

## Results

### Increased DOX-PMs-NPMBP Sensitivity in ALL Cell Lines and Primary Cells Compared to DOX

We determined whether DOX-PMs-NPMBP may effectively inhibit cell growth in DOX-resistant cell lines (Nalm6/DOX cells and Nalm6-Luc/DOX cells) and their parental cell lines (Nalm6 cells and Nalm6-Luc cells). Overall, ALL cells were more sensitive to DOX-PMs-NPMBP compared with DOX (Figure 1D, E). Although the average IC50 value of DOX in resistant Nalm6 cells was around 20-fold higher than that of DOX in its parental cells, cell growth was significantly suppressed by DOX-PMs-NPMBP with a dose-dependent way. The average IC50 value of DOX-PMs-NPMBP was 0.08 ± 0.01 µg/ml and 0.62 ± 0.25 µg/ml in Nalm6-Luc cells and corresponding DOX-resistant cells, respectively, reflecting a 2.6- and 7.0-fold reduction compared to DOX alone. Similar results were found in Nalm6 cells and Nalm6/DOX cells. The average IC50 value of DOX-PMs-NPMBP was 3.5-fold lower than that of DOX in Nalm6 cells and Nalm6/DOX cells. Interestingly, DOX-PMs-NPMBP decreased cell proliferation more significantly than the effects of DOX alone in primary ALL cells. Primary ALL cells from newly diagnosed or relapsed patients, were more sensitive to DOX-PMs-NPMBP compared with DOX. The average IC50 value was significantly decreased from 1.92 ± 0.33 µg/ml and 2.98 ± 0.46 µg/ml in DOX group to 1.18 ± 0.23 µg/ml and 1.42 ± 0.12 µg/ml in DOX-PMs-NPMBP group in newly diagnosed and relapsed patients, respectively. Noticeably, as shown in Figure 1C, the blank PMs without NPMBP and DOX showed no significant toxicity in either Nalm6 cells or Nalm6/DOX cells. About 85% cell viability was observed at maximum polymer concentrations equivalent to 40 µg/ml DOX.

### DOX-PMs-NPMBP Induces Apoptosis in DOX-Resistant Nalm6 Cells and DOX-Sensitive Nalm6 Cells

**FIGURE 2:**
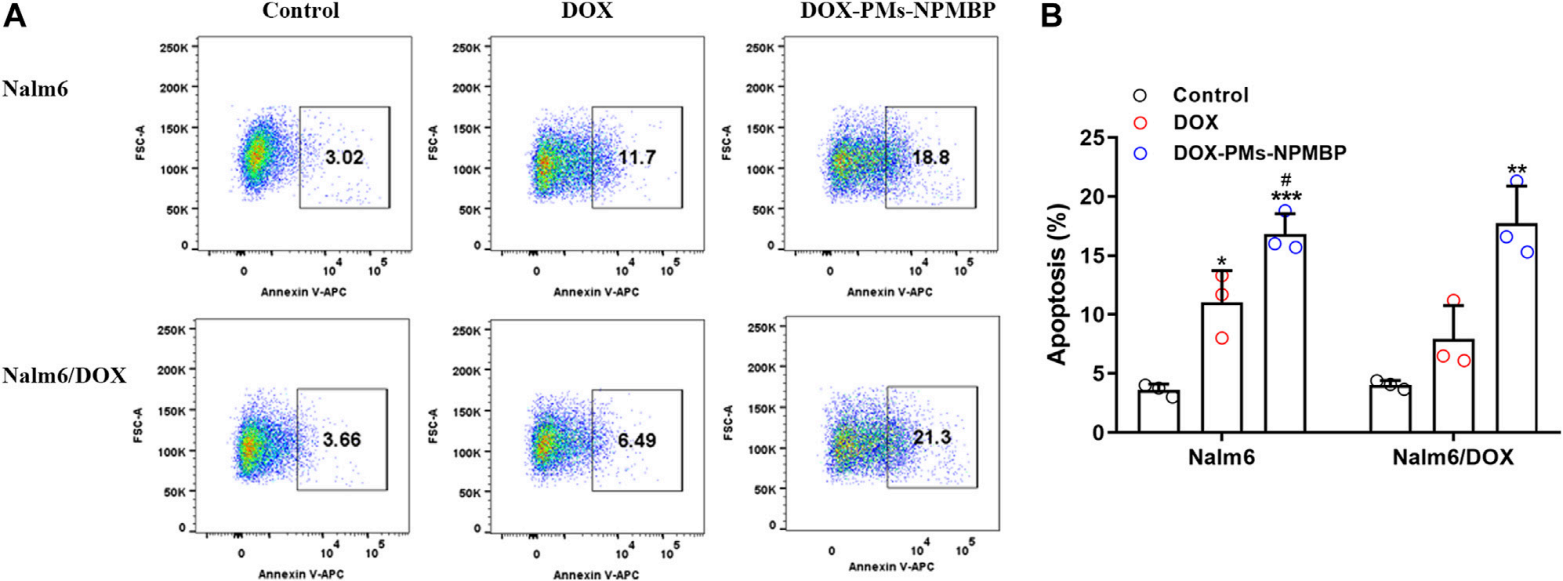
DOX-PMs-NPMBP induces apoptosis in Nalm6/DOX cells. Nalm6/DOX cells were incubated with DOX or DOX-PMs-NPMBP for 24 h. Frequency of cells undergoing apoptosis was stained with Annexin V-APC and analyzed by flow cytometry. **(A)** Representative flow-cytograms of Annexin V^+^ cells in each groups **(B)** Summary data for the frequency of apoptotic cells in each measured as in panel A. Data are generated from three independent experiments. *p < 0.05, **p < 0.01, ***p < 0.001 compared with control, ^#^ *p* < 0.05 compared with DOX.

We next assessed the role of DOX-PMs-NPMBP on apoptosis induction in Nalm6 cells and Nalm6/DOX cells*.* The results demonstrated that DOX-PMs-NPMBP induced apoptosis in both cell lines compared to DOX treatment alone (Figure 2). When treated with DOX-PMs-NPMBP for 24 h, the ratio of apoptotic cells was 16.8 ± 1.7 and 17.7 ± 3.2%, respectively, reflecting a 1.5-fold and 2.2-fold increase compared to Nalm6 cells and Nalm6/DOX cells following single DOX administration. These results indicate that DOX-PMs-NPMBP can increase DOX chemosensitivity in either DOX-responsive or DOX-resistant ALL cells.

### DOX-PMs-NPMBP promotes the intracellular retention of DOX in DOX-resistant Nalm6 cells

**FIGURE 3:**
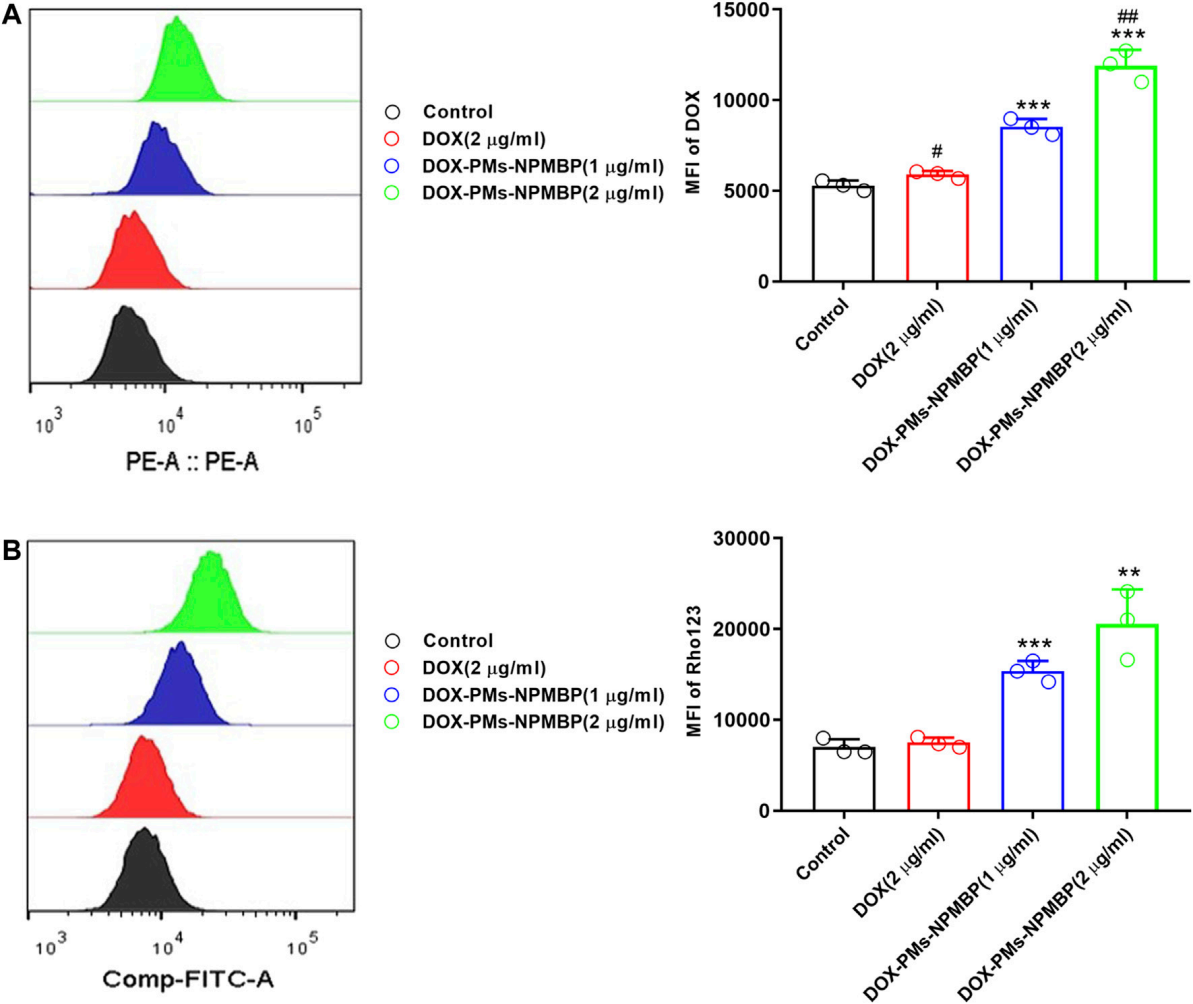
DOX-PMs-NPMBP promotes intracellular accumulation of DOX or Rhodamine123 (Rho123) in Nalm6/DOX cells. Nalm6/DOX cells were treated with indicated concentration of DOX and DOX-PMs-NPMB for 12 h. The intracellular retention of DOX was evaluated by flow cytometry**(A)**. For the drug efflux ability assay, Rho123 at 2 μM was added to each well above and incubated for another 30 min at 37°C. After washed, flow cytometer analysis was used to assess the MFI of Rho123 in the Nalm6/ADR cells through FITC channel **(B)**. Data reflect the mean ± SD of three separate experiments. MFI: mean fluorescence intensity, **p < 0.01, ***p < 0.001 compared with DOX, ^#^ *p* < 0.05 compared with control, ^##^ *p* < 0.01 compared with DOX-PMs-NPMBP (1 µg/ml).

To illustrate the mechanisms of DOX-PMs-NPMBP growth inhibitory effect in drug-resistant Nalm6 cells, we monitored the intracellular accumulation of DOX after treatment. As shown in Figure 3A, it showed slightly increased the intracellular retention of DOX in Nalm6/DOX cells after treated with DOX. The MFI of DOX significantly increased in a dose-dependent manner in Nalm6/DOX cells after DOX-PMs-NPMBP administration. Compared with DOX alone, DOX uptake was dramatically increased by 1.4- and 2.0-fold in Nalm6/DOX cells, after 1.0 and 2.0 µg/ml DOX-PMs-NPMBP treatment, respectively.

### Biological evaluation shows significant accumulation of intracellular Rho123 in the Nalm6/DOX cells upon DOX-PMs-NPMBP administration

Flow cytometry analysis showed that the MFI of Rho123 was not significant different to that of untreated control in Nalm6/DOX cells after treatment with DOX at 2.0 µg/ml (*p* = 0.4419), In contrast, DOX-PMs-NPMBP-treated resistant Nalm6/DOX cells dose-dependently increased intracellular accumulation of Rho123. As compared to DOX alone, the intracellular retention of Rho123 was significantly increased by 2.1- and 2.9-fold in Nalm6/DOX cells, after 1.0 and 2.0 µg/ml DOX-PMs-NPMBP treatment, respectively (Figure 3B).

### Bioinformation Analysis Shows Significant Transcriptional Changes in Nalm6/DOX cells Following DOX-PMs-NPMBP Treatment

**FIGURE 4:**
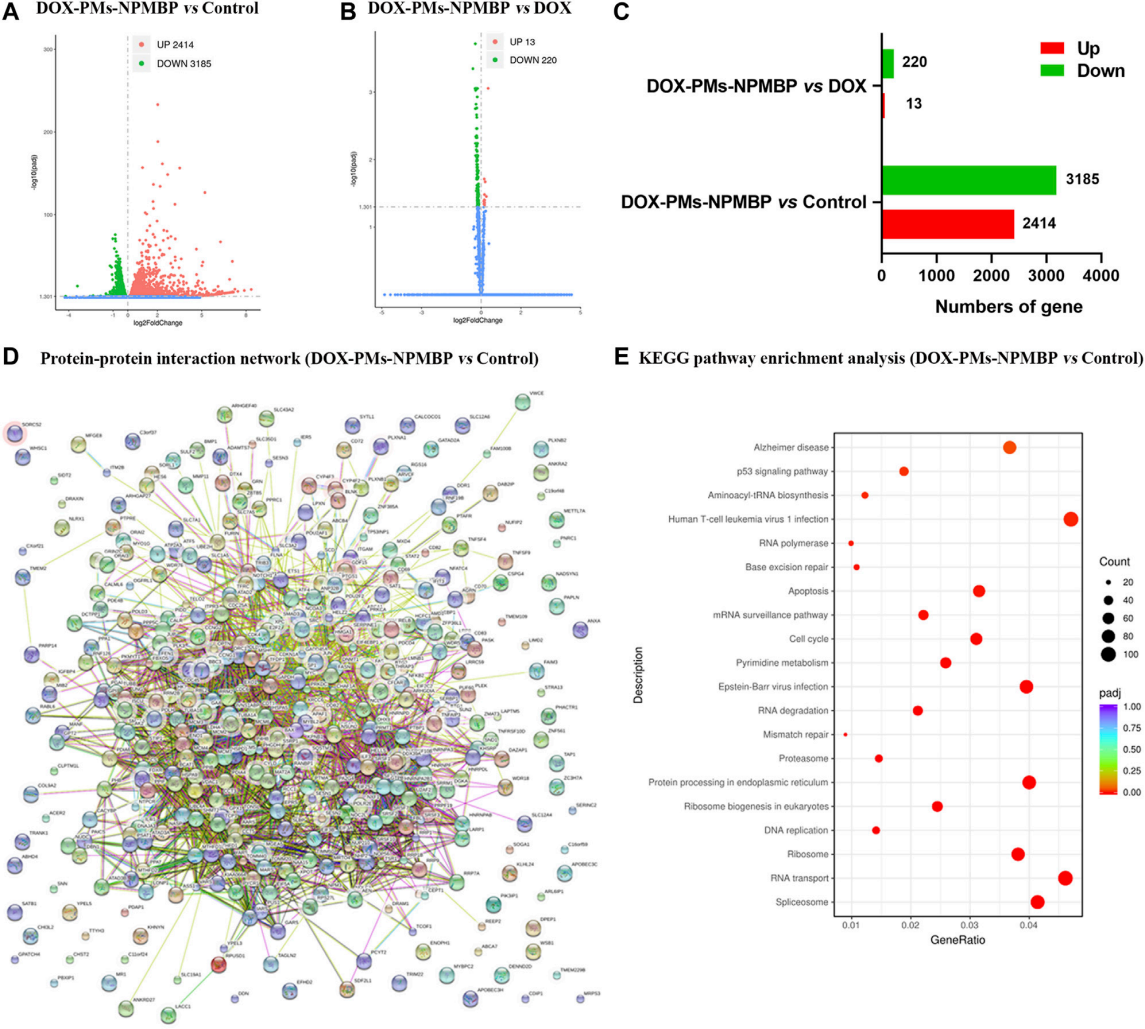
Bioinformation analysis based on RNA-seq data. **(A–C)** Volcano plots of differentially expressed genes (DEGs) in Nalm6/DOX cells administered DOX, DOX-PMs-NPMBP, and vehicle control. **(D)** DOX-PMs-NPMBP-associated protein interaction network. Each circle means a protein/gene (node). **(E)**DOX-PMs-NPMBP-associated Kyoto Encyclopedia of Genes and Genomes (KEGG) pathway enrichment analysis.

To better understand the mechanisms driving DOX-PM-NPMBP increased anti-leukemia effects, we compared the transcriptional profiles of Nalm6/DOX cells following treatment with either DOX-PMs-NPMBP or DOX. Figures 4A–C show volcano plot of differential gene expressions (DEGs) in DOX-PMs-NPMBP-treated cells vs. vehicle control cells. We identified 2,414 upregulated genes, which are displayed as red dots, and 3,185 downregulated genes, which are displayed as green dots, between the vehicle control and the DOX-PMs-NPMBP treatment. We found 13 genes that were upregulated and 220 genes that were downregulated when compared DOX vs. DOX-PMs-NPMBP treatments. Next, we constructed the protein-protein interaction (PPI) and DEG network *via* the Search Tool for the Retrieval of Interacting Genes (STRING) repository (https://string-db.org/). The STRING diagram for the initial cohort of 400 gene proteins is shown in Figure 4D. This analysis displayed a clustering coefficient of 0.524 and a PPI enrichment *p*-value of 0. This suggests adequate connectivity and acceptable conclusions for analysis of the networks. To gain insight into the potential pathways that might be specifically associated with increased DOX-PMs-NPMBP-efficacy in Nalm6/DOX cells, we performed Kyoto Encyclopedia of Genes and Genomes (KEGG) enrichment analysis among different treatment groups. For DOX-PMs-NPMBP nanoparticle vs. vehicle control, 21 pathways were identified (*p* < 0.05, Figure 4E). Among those, p53 protein (gene symbol TP53) overlapped five pathways, e.g., Epstein-Barr virus infection, cell cycle, apoptosis, human T-cell leukemia virus one infection and p53 signaling pathway. The RNA-seq data regarding this study are available on the NCBI website database *via* the link: https://www.ncbi.nlm.nih.gov/Traces/study/?acc=PRJNA668604.

**FIGURE 5:**
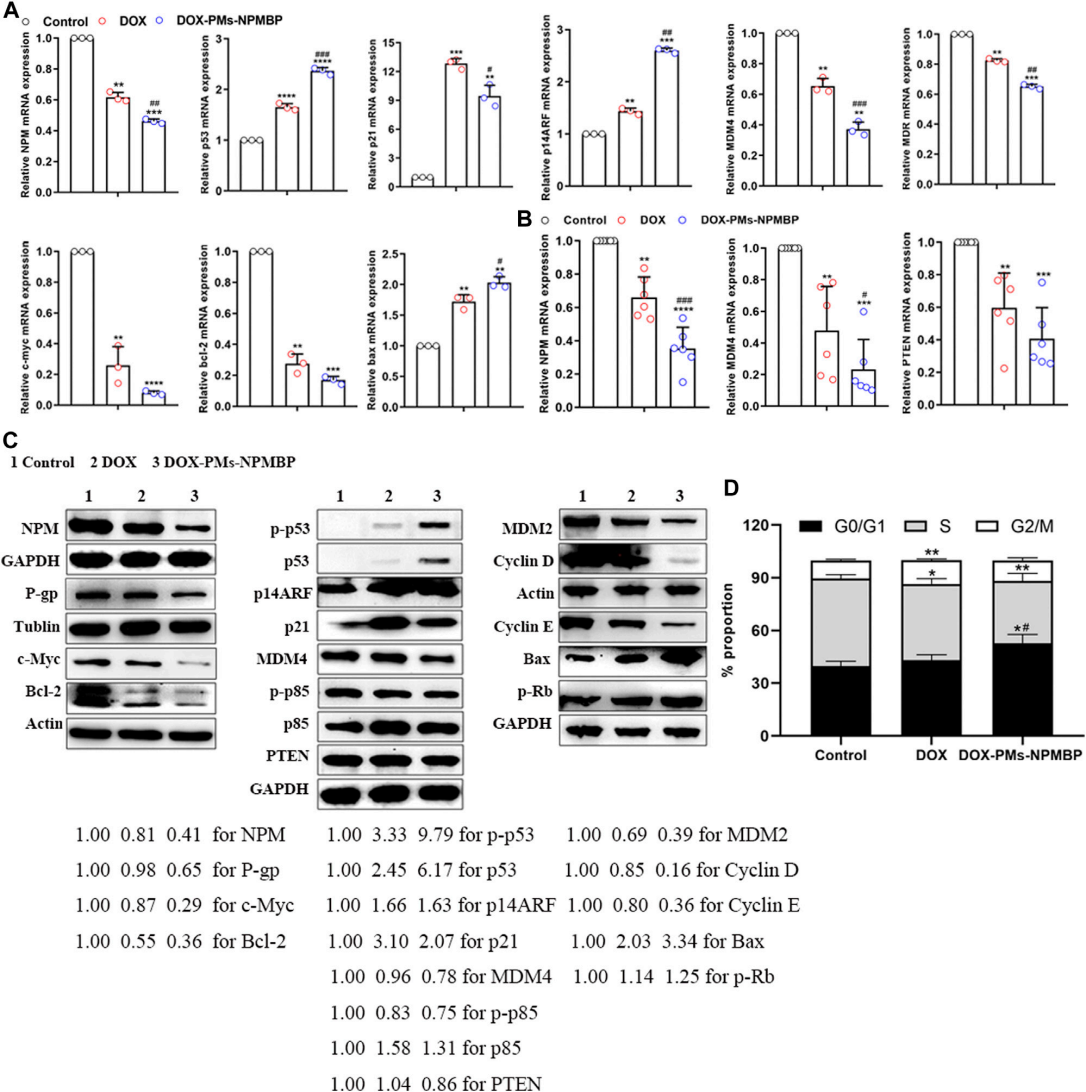
Effects of DOX-PMs-NPMBP on mRNA and protein expression levels, and cell cycle distribution. Nalm6/DOX cells **(A)** and primary ALL cells from patients **(B)** were treated with DOX or DOX-PMs-NPMBP for 12 h, cells were harvested, and total RNA was isolated. Real-time PCR analysis was performed. All assays were repeated three times. **p < 0.01, ***p < 0.001, ****p < 0.0001 compared with control, ^#^ *p* < 0.05, ^##^ *p* < 0.01, ^###^ *p* < 0.001 compared with DOX. **(C)** Protein expression levels in the Nalm6/DOX cells treated with DOX or DOX-PMs-NPMBP for 24 h as measured by western blotting. Tublin, Actin or GAPDH was employed as an internal reference. The experiments were repeated at least three times. The intensity of different protein bands was quantified and normalized with GAPDH, Tublin or Actin. Changes in protein expression levels are represented as percentage change from untreated control levels, which were set to 100% (1.00). **(D)** Cell cycle distribution analysis of Nalm6-Luc/DOX cells administered DOX or DOX-PMs-NPMBP for 24 h. Data are mean ± SD of three experiments performed independently. *p < 0.05, **p < 0.01 vs. control. ^#^ *p* < 0.05 vs. DOX.

RNA-seq data was further validated by qRT-PCR. The relative gene expression levels of specific targets in Nalm6/DOX cells and primary ALL cells from patients were quantified (Figures 5A, B). Compared with either DOX or vehicle control, DOX-PMs-NPMBP conditioning significantly downregulated the mRNA levels of NPM, MDR, c-myc, bcl-2, MDM4 and PTEN*,* and markedly upregulated p53*,* bax, p21 and p14ARF. In line with the changes of mRNA expression after DOX-PMs-NPMBP treatment, we found that the protein expression of NPM, MDR, c-Myc, Bcl-2, MDM4 and PTEN dramatically decreased, while the p53, phospho-p53, Bax, p21 and p14ARF protein remarkably increased in the cells after administered DOX-PMs-NPMBP. In addition, DOX-PMs-NPMBP induced the protein expression of p85 and phospho-Rb, but reduced the levels of MDM2, phospho-p85, cyclin D, cyclin E in Nalm6/DOX cells (Figure 5C).

### DOX-PMs-NPMBP Induces the G0/G1 Cell Cycle Arrest in Nalm6/DOX Cells

We further explored the effects of DOX-PMs-NPMBP on cell cycle distribution of Nalm6/DOX cells. Compared to the control group, DOX-PMs-NPMBP remarkably increased the frequency of cells in the G0/G1 phase (52.82 ± 4.96% *vs* 39.76 ± 2.75%, *p* = 0.016), while decreased the proportion of cells in the S phase (35.38 ± 4.33% *vs* 49.91 ± 2.20%, *p* = 0.007). In contrast, DOX alone slightly reduced cell amounts in the S phase, and increased frequency of G2/M phase cells. Moreover, the proportion of G0/G1 phase cells upon DOX-PMs-NPMBP administration was significantly increased in comparison with the DOX value (52.82 ± 4.96% *vs* 43.11 ± 3.11%, *p* = 0.045, Figure 5D).

### DOX-PMs-NPMBP Inhibits the Growth of Implanted Nalm6-Luc/DOX Cells in BALB/C-Nude Mice

**FIGURE 6:**
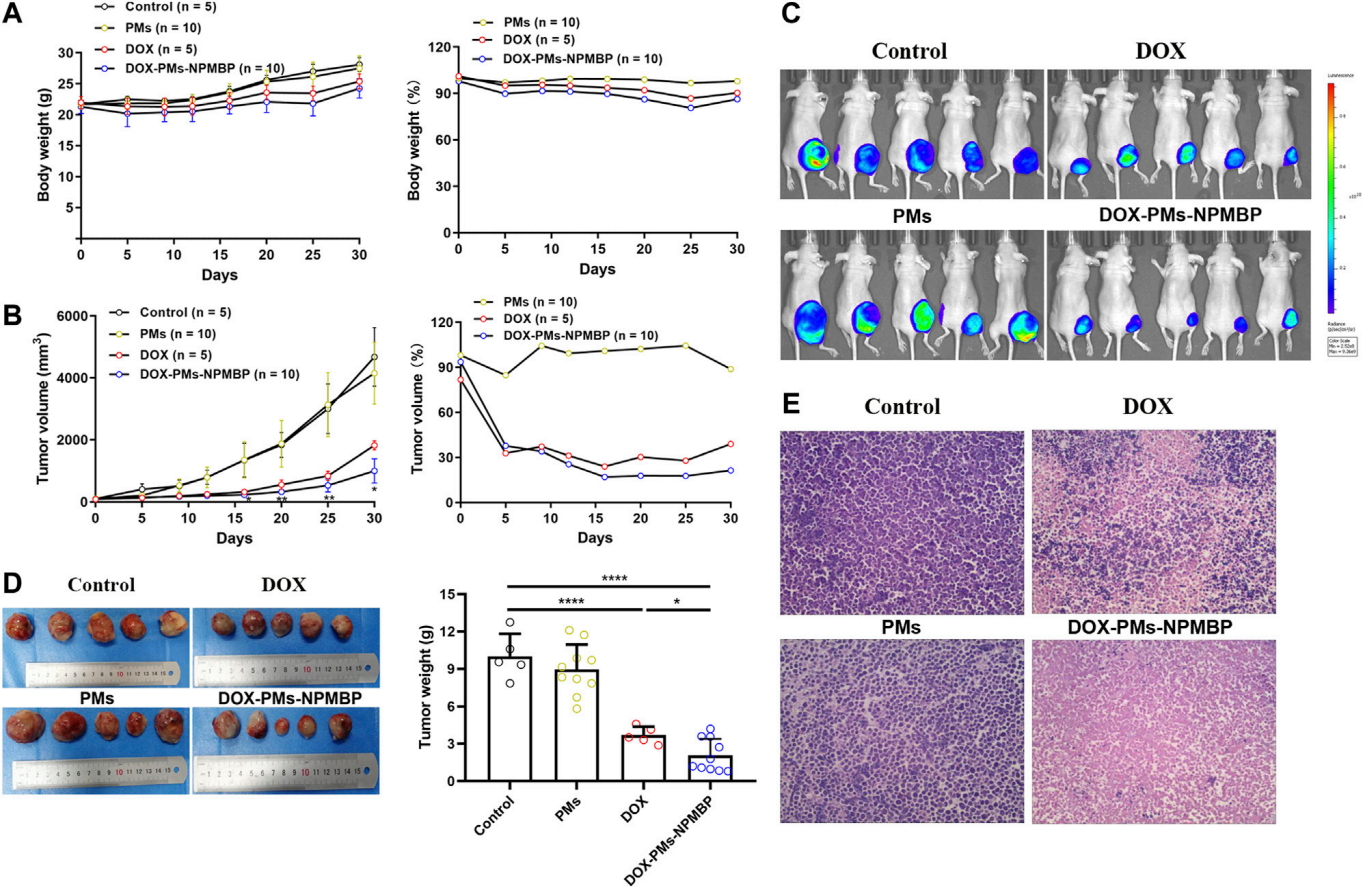
DOX-PMs-NPMBP inhibits xenograft growth and enhances antileukemia activity in Nalm6-Luc/DOX xenograft nude mice. The nude mice with Nalm6-Luc/DOX xenografts were randomly divided into four groups and intraperitoneally administered DOX, DOX-PMs-NPMBP, PMs, or PBS once daily for 3 days. Body weights and tumor size were measured at the indicated time scheme by a caliper (tumor volume = shortest diameter^2^ × longest diameter/2). **(A)** Body weight during follow-up. The relative levels of body weight are represented as percentage changes from vehicle control levels, which were set at 100%. **(B)** Tumor volume at each time point. The relative levels of tumor volume are represented as percentage changes from vehicle control levels, which were set to 100%. *p < 0.05, **p < 0.01 vs. DOX group. **(C)** Representative of whole-body luciferase reporter images from vehicle, DOX or DOX-PMs-NPMBP treated mice. **(D)** Representative of photographs of tumors excised from xenograft mice after completed the study. The tumor weight was measured. *p < 0.05, ****p < 0.0001. **(E)** Hematoxylin-eosin-stained sections of tumor tissue from representative control and treated mice.

To assess the impact of DOX-PMs-NPMBP *in vivo*, this compound was tested in Nalm6-Luc/DOX subcutaneous transplant model in BALB/C-nude mouse. Either DOX or DOX-PMs-NPMBP regimen was well tolerated when administered at 3 mg/kg once daily for 3 days during the follow-up. As shown in Figure 6A, the body weights in each group increased slightly with time after treatment. When normalized the body weight of PBS control group, the relative body weight in PMs group was similar to that of the controls. The relative body weight in both DOX- and DOX-PMs-NPMBP-treated group was slightly lower than that in PMs group; but without significant differences between the two regimens.

The average tumor volume in the DOX-treated animals was obviously smaller than that of PBS- and PMs-conditioned mice, but larger than that of DOX-PMs-NPMBP-administered recipients (Figure 6B). Animals bearing subcutaneous Nalm6-Luc/DOX tumor were imaged by IVIS SPECTRUM Imaging System pre- and post-initial treatment. Bioluminescent imaging demonstrated that Nalm6-Luc/DOX xenograft mice presented strong therapeutic response to DOX-PMs-NPMBP. Lower tumor burden was observed in the recipients which received DOX-PMs-NPMBP as compared to that DOX alone (Figure 6C). Following 30 days after initial regimen, DOX-PMs-NPMBP group demonstrated an excellent antitumor activity with the inhibition rate of 79.2% compared to vehicle control, which remained the lowest among the three groups (Figures 6C,D).

Histologically, xenograft tumor in control group was characterized by high homogeneity and integrity, and unnormal proliferation state; Nalm6-Luc/DOX cells found in the vehicle control’s subcutaneous tumor tissues were different sizes with the large stained nuclei, and were of a round shape. In contrast, the tumor sections of DOX and DOX-PMs-NPMBP showed different levels of inflammation and cell necrosis, depicted by a heterogeneous dyeing and messy cell morphology. Of note, less tumor cells, more erythrocytes and fibroblast cells were visible in the tumor tissues from DOX-PMs-NPMBP-treated mice (Figure 6E).

### DOX-PMs-NPMBP Enhances Antileukemia Activity in the NCG Nalm6-Luc/DOX Transplant Models

**FIGURE 7:**
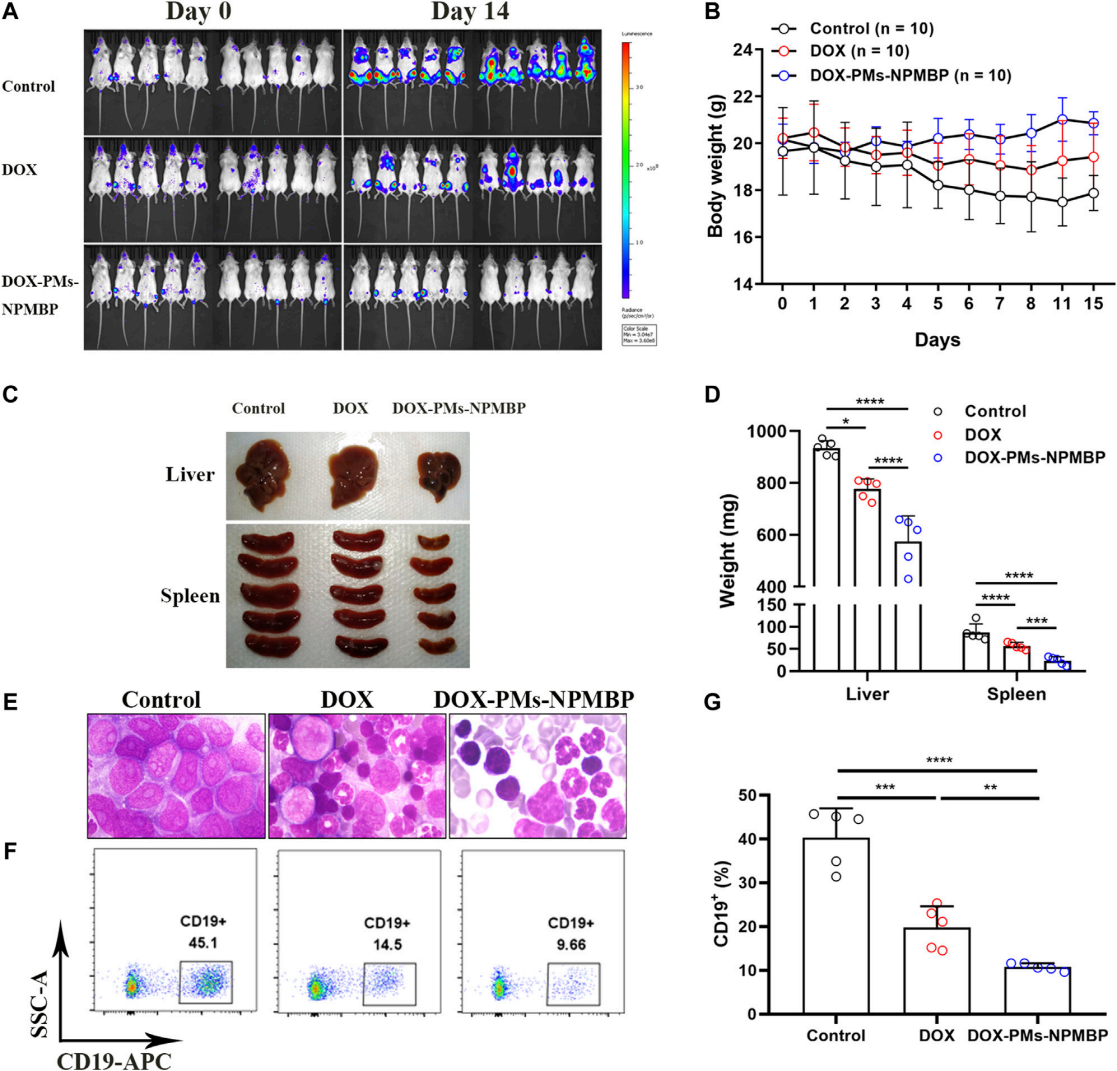
DOX-PMs-NPMBP enhances antileukemia activity in NOD-Prkdc^em26^Il2rg^em26^Nju (NCG) mice implanted with Nalm6-Luc/DOX cells. A total of 1.0 × 10^4^ Nalm6-Luc/DOX cells were infused into NCG mice by tail vein injection. Seven days later, mice were intraperitoneally administered daily single dose of DOX, DOX-PMs-NPMBP, or PBS for 3 days. The day before initial regimen was designated as day 0. Animals were euthanized at the 14th day after the initial treatment. **(A)** Bioluminescence images of the ventral/dorsal area taken at pre- and post-treatment. **(B)** Body weights at each time point. **(C)** Representative photographs of liver and spleen excised from implanted NCG mice. **(D)** Liver and spleen weight changes in different conditioning group mice. **(E)** Representative Wright-Giemsa-stained sections of harvested bone marrow (BM) cells in each group mice. **(F)** Representative images of flow-cytogram illustrating the CD19^+^ Nalm6-Luc/DOX cells in BM. Dot plots reflect CD19-APC vs. side scatters. *p < 0.05, ***p < 0.001, ****p < 0.0001. **(G)** The percentages of CD19^+^ cells in BM were measured by flow cytometry. **p < 0.01, ***p < 0.001, ****p < 0.0001.

We further assessed the *in vivo* anti-leukemia activity of DOX-PMs-NPMBP in NCG mice. A total of 1.0 × 10^4^ Nalm6-Luc/DOX cells were infused into NGG mice by tail vein injection. Seven days later, the mice were administered a single dose of DOX or DOX-PMs-NPMBP daily for 3 days. The day before initial regimen was designated as day 0. As shown in Figure 7A, DOX-PMs-NPMBP showed significant anti-leukemia activity, with less fluorescence signals 14 days after initial administration. In contrast, animals from vehicle control or DOX alone presented strong fluorescence signals, which widely spread to different organs. Body weights from each group mice were also monitored during the follow-up. Significant decreased body weight could be observed in control group; however, there was no significant weight loss for DOX- and DOX-PMs-NPMBP-treated groups. Instead, the body weight of the mice from the nanoparticle conditioning group increased slightly during the study period, indicating the novel drug delivery system produced less systematic toxicity and reached higher therapeutic effect (Figure 7B). Moreover, larger liver and spleen were found in animals from either control group or DOX group compared to DOX-PMs-NPMBP-treatment (Figures 7C, D). Wright-Giemsa staining results demonstrated increased immature blast cells in the BM from vehicle control mice and DOX-treated mice. However, BM samples from DOX-PMs-NPMBP-conditioned mice showed more mature myeloid cells and lymphocytes (Figure 7E). Nalm6-Luc/DOX cells in the BM from individual mice were further confirmed by flow cytometric analysis. As shown in Figures 7F,G, the frequency of CD19^+^ Nalm6-Luc/DOX cells was dramatically decreased upon DOX-PMs-NPMBP administration in comparison to either vehicle control or DOX-only treatment (*p* < 0.0001 *vs* control, *p* < 0.01 *vs* DOX).

### DOX-PMs-NPMBP Improves the Survival of Nalm6-Luc/DOX Xenograft NCG Mice

**FIGURE 8:**
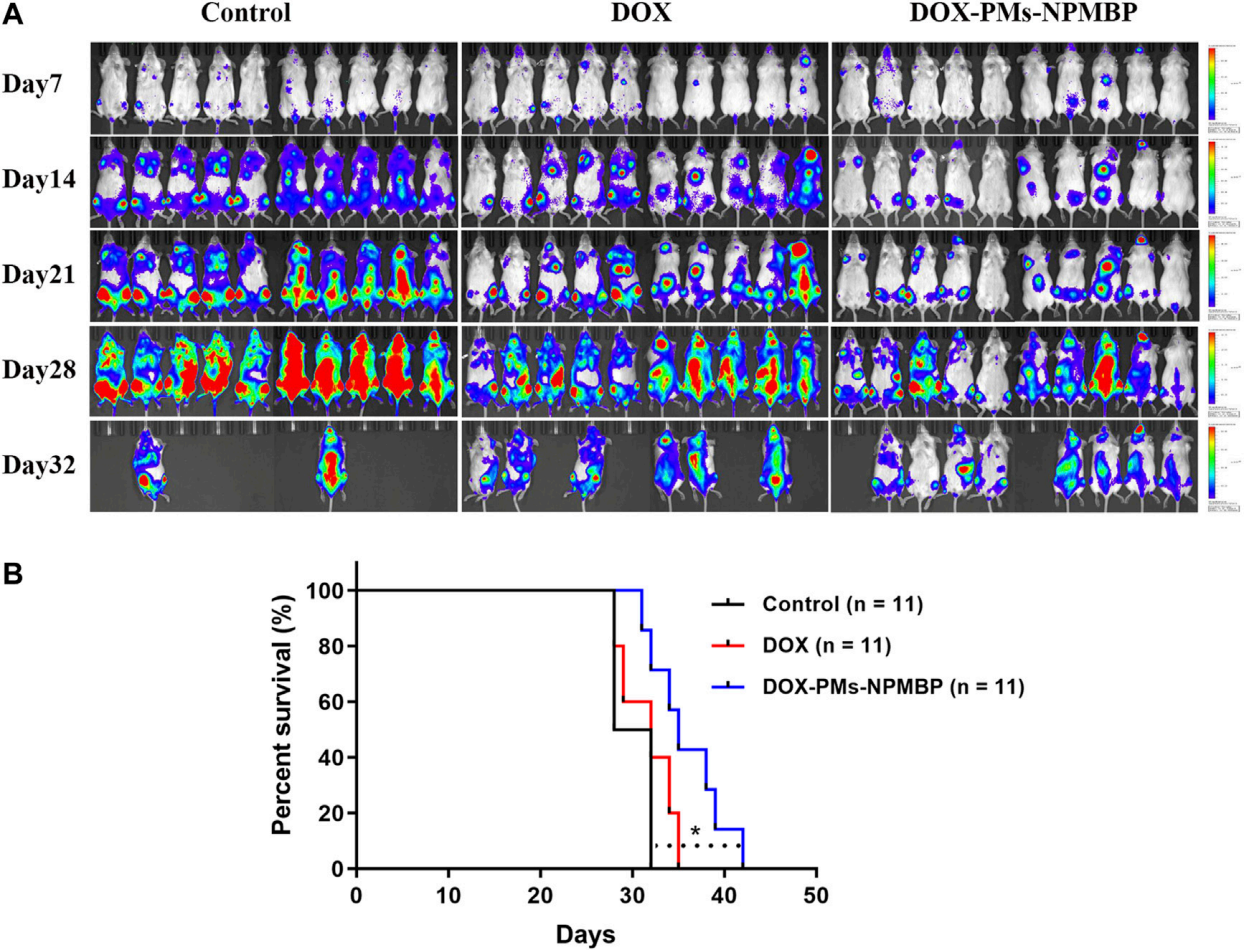
DOX-PMs-NPMBP improves survival in NCG mice implanted with Nalm6-Luc/DOX cells. The day of implantation with Nalm6-Luc/DOX cells was designated as day 0. ALL xenograft models were monitored during the study period. Survival time of the recipients were recorded. **(A)** Representative bioluminescence images of the ventral/dorsal area taken on different time points during follow-up. **(B)** Kaplan-Meier survival curves. *p < 0.05 compared with control.

The overall survival of the animals with the different regimens was monitored. *In vivo*, administration of DOX in Nalm6-Luc/DOX cells yielded only partial improvement in overall survival, through partial delay but incomplete prevention of leukemia cell growth (*p* > 0.05 compared to PBS vehicle control). However, DOX-PMs-NPMBP regimen significantly reduced Nalm6-Luc/DOX leukemia burden and improved overall survival as compared with the control (*p* < 0.05). Thus, the *in vivo* results further confirm the enhanced therapeutic efficacy of DOX-PMs-NPMBP for resistant ALL (Figure 8).

## Discussion

ALL is a hematologic malignant disease that occurs due to a buildup of lymphoid progenitor cells in various tissues, particularly bone marrow. The rate at which it is cured in adult ALL patients is between 20 and 40%, provided they undergo chemotherapy (Sive et al., 2012; Richard-Carpentier et al., 2019). There has been a significant increase in our collective understanding of the pathogenesis of ALL over the past decade, as well as novel approaches for treatment. In particularly, the administration of monoclonal antibodies, CAR-T cell therapies, and other novel targeted strategies in ALL show tremendous potential to improve cure and survival rates. For example, Inotuzumab ozogamicin is an anti-CD22 antibody drug conjugate that is set to deliver cytotoxic drugs to B-ALL cells expressing CD22, and shown great outcomes in patients with relapsed or refractory ALL (Kantarjian et al., 2016). We previously confirmed that NPM overexpression was involved in MDR, and was associated with the prognosis of ALL (Hu et al., 2011; Lin et al., 2013; Wang et al., 2015). Engineered nanoparticles have certain chemical and physical characteristics that give them especially useful medical purposes. As such, in the present study, the antileukemia activity of NPM binding protein- and DOX-conjugated nanoparticle in ALL cells were evaluated, and the underlying mechanisms were fully elucidated.

Nanoparticles have high permeability and retention, allowing them to deliver drugs that can accumulate within the tumor (Markman et al., 2013). DOX-PMs-NPMBP is a novel method of delivery, in which adding PEG has refined the polymers. This avoids opsonization and increases *in vivo* circulation time. Nanoparticles also have wide surface areas, which provides ample space for the absorbed protein NPMBP to attach. Additionally, nanospheres have solid cores, which are optimal for the attachment of chemotherapeutic agent DOX. Moreover, the water-soluble polymer PLA can covalently bind to DOX, which raises the time of circulation and lowers toxicity in normal tissues (Markman et al., 2013). Furthermore, PLA is a polymeric biodegradable nanoparticle that can be used to create nanomedicines that have been approved by the Food and Drug Administration (FDA) in the United States (Mattheolabakis et al., 2012), which further inspired us to carry out an extensive investigation on the effects of the novel drug delivery system DOX-PMs-NPMBP in ALL cells.

Recently, Zhang and colleagues reported that Saporin, a strong cytotoxic compound, can significantly slow the growth of parental cancer cells and resistant ATP-binding cassette (ABC) transporter subfamily B member 1 (ABCB1)- and ABC transporter subfamily G member 2 (ABCG2)-overexpression cells when it has been encapsulated in lipid-based nanoparticles (Zhang et al., 2020). We found the resistant ALL cell lines, as well as the primary ALL cells from relapsed patients, were more sensitive to DOX/NPMBP-conjugated nanoparticles DOX-PMs-NPMBP. Although the resistant Nalm6/DOX cells showed a little effect in response to DOX-only administration, however, more than 2-fold increase in the frequency of apoptotic cells was observed in those cells responding to DOX-PMs-NPMBP vs. DOX alone. Consistently, a higher frequency of DOX-resistant ALL cells undergo G0/G1 phase arrest upon DOX-PMs-NPMBP administration. These observations suggest that DOX and NPMBP conjugation into nanoparticles holds significant potential for enhancing DOX bioavailability and overcoming drug resistance in ALL cells.

When the gene expression profiles of Nalm6/DOX cells were analyzed comprehensively by RNA-seq technology, KEGG enrichment analysis of DEGs showed that 21 pathways were significantly different in following DOX-PMs-NPMBP treatment. Of note, TP53 overlapped in the five different pathways when compared the difference between DOX-PMs-NPMBP vs. vehicle control. And KEGG analysis didn’t show that TP53 were significantly enriched in different biological process between DOX-PMs-NPMBP vs. DOX treatment (Supplementary Figure 2 **,** https://www.ncbi.nlm.nih.gov/Traces/study/?acc=PRJNA668604), exposing p53 as an essential player in DOX-PMs-NPMBP biological activity. Previous studies revealed that the activation of p53 induces several protective reaction activities, such as cell cycle arrest, senescence or cell death. However, p53 inactivation, which is usually caused by TP53 gene mutation or negative regulation of wild type (wt) protein product, is commonly found in most human cancer (Ding et al., 2020; Jiang et al., 2020; Long et al., 2020). More recently, pharmacological manipulation of p53 has been a highly attractive strategy in the development of new anticancer treatments (Lazo, 2017; Duffy et al., 2020). Thus, the present study may be of great value to investigate the underlying mechanisms of DOX-PMs-NPMBP in the pre-B Nalm6 cells, which typically harbor with wt p53.

Furthermore, the underlying mechanisms of DOX-PMs-NPMBP-enhanced anti-leukemia and cell death effects were explored. DOX-PMs-NPMBP dramatically inhibited MDR as well as the targeting molecule NPM as compared to either DOX or vehicle control. Nanoparticle carrier has been reported to circumvent P-gp-mediated resistance, enhancing cellular drug-uptake through the forming of ion pairs and endocytosis, the lowering of ATP, regulating the function and expression of P-gp, as well as alterations in the P-gp downstream signaling pathways (Dong and Mumper, 2010; Zhang et al., 2020). This is a likely explanation for higher level of intracellular accumulation of DOX in the resistant cells when conditioned with DOX-PMs-NPMBP. Moreover, Rho123 efflux assay, a very sensitive functional assay for ABC-transporter P-gp, was further included in this study. The novel DOX-PMs-NPMBP nanoparticle system could markedly modulate the functional activity of P-gp, thereby reducing the drug pumping P-gp-mediated efflux of Rho123 in the resistant ALL cells in comparison to DOX, suggesting the classical MDR phenotype-dependent mechanism was involved in the action of DOX-PMs-NPMBP on DOX-resistant cells.

NPM plays important role for the nucleolar localization of oncogenic c-Myc and enhances c-Myc transformation. Constitutive NPM overexpression may stimulate c-Myc-mediated rRNA synthesis (Li and Hann, 2013). In line with these reports, our data delineates that DOX-PMs-NPMBP not only completely abolished the expression of c-Myc, but also markedly induced the activation of p14ARF, an important partner of NPM and a major tumor suppressor. P14ARF also has functions relating to nucleolar processes, as well as cell cycle arrest, *via* the MDM-p53 pathway (Lo et al., 2015). Additionally, NPM has been known to play key roles in maintaining p53 stability and regulating its transcriptional activation (Colombo et al., 2002). Interestingly, the presence of DOX-PMs-NPMBP in Nalm6/DOX cells results in the robust activation of p53 and cyclin-dependent kinase inhibitor one p21 coupled with the greatly suppression of MDM2 and MDM4, the two key negative regulators of p53 (Jackson-Weaver et al., 2020; Li and Lozano, 2013). Previous study showed that p21 activation blocked cell cycle progression by function as the inhibitor of cyclin D/E kinases, which are required for the G1-S phase transition of cell cycle (Aubrey et al., 2018; Karimian et al., 2016; Kim and Choi, 2020). Thus, it is likely that we found the exposure of DOX-PMs-NPMBP markedly downregulated the expressions of cyclin D and cyclin E. Moreover, Hüllein J *et al* previously demonstrated the key role for tumor suppressor p53 in Burkitt lymphoma (BL) and identified MDM4 as a therapeutic target in various cancers. MDM4 knockdown activated p53, induced cell-cycle arrest, and lowered the growth of tumor in a xenograft model (Hullein et al., 2019). Recently, pharmaceutical inhibitors targeting MDM2 and MDM4 have been explored to reactive the p53/Rb tumor suppressor function; and the preliminary response data from the clinical trial in acute myeloid leukemia patients have been gaining increasing interest (Burgess et al., 2016; Vu et al., 2020). Likewise, our findings give an insight into the DOX-PMs-NPMBP-induced MDM2 and MDM4 dual inhibition, which might put forward the clinical perspectives of DOX-PMs-NPMBP to ALL therapy.

**FIGURE 9:**
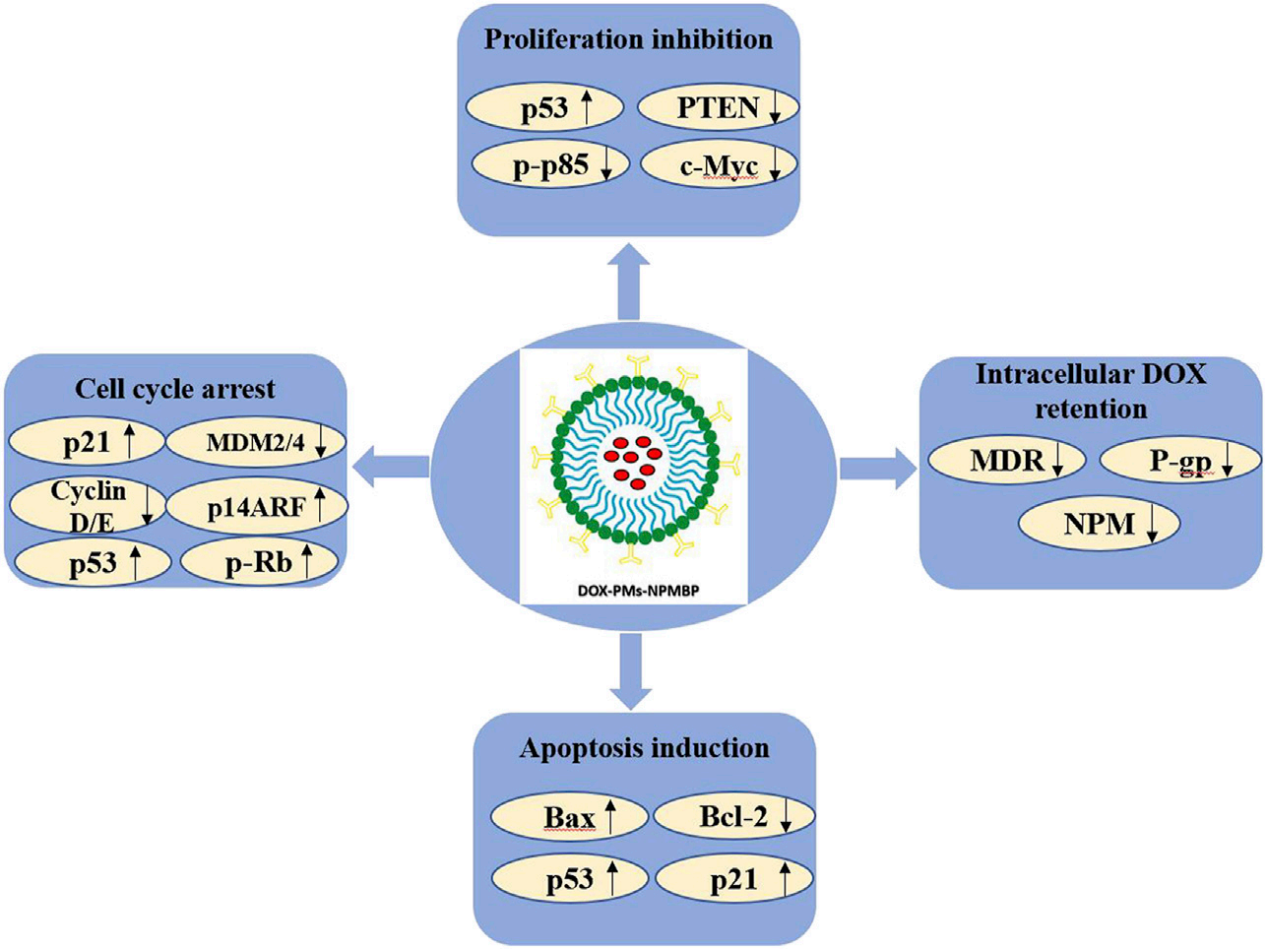
The molecular mechanisms linking the anti-leukemia activity of DOX-PMs-NPMBP in DOX-resistant ALL cells.

Nevertheless, compared with control or DOX alone, we found DOX-PMs-NPMBP-induced significant activation of p53, coinciding with the inhibition of PTEN as well as phospho-p85 and the PI3K regulatory subunit. PTEN is the negative regulator of PI3K/Akt signaling pathway, which is frequently activated in various cancer cells and associated with chemoresistance (Chen et al., 2014; Li et al., 2015; Chen et al., 2020; Yang et al., 2020). Along these lines, we observed the enhanced inhibitory effects on the anti-apoptotic Bcl-2, and the subsequent induction of proapoptotic Bax by DOX-PMs-NPMBP. Taken together and as the most straight forward interpretation of our data, we suggest that treatment with DOX-PMs-NPMBP-induced effects on cell viability mainly due to activated p53, leading to the subsequent p53-mediated apoptosis as well as cell cycle arrest. The graphical depiction regarding the molecular mechanisms of DOX-PMs-NPMBP working on resistant ALL cells were showed in Figure 9.

Nanoparticle-based therapy may offer multiple advantages over conventional therapeutics in various cancers, *e.g.* high carrier capacity, targeted delivery and therapy, improvement of biological properties, and reversal of MDR (Dong and Mumper, 2010; Sanna et al., 2014; Zhang et al., 2020). The therapeutic properties of DOX-PMs-NPMBP were further evaluated in a Nalm6-Luc/DOX xenograft BALB/C-nude and NCG ALL models. Lower tumor burden was observed in DOX-PMs-NPMBP-treated recipients as compared to the animals administered with DOX alone. Tumor inhibition rate achieved as high as 79.2% in DOX-PMs-NPMBP group when finished the following-up. Of note, the regimen of DOX-PMs-NPMBP significantly prolonged mouse survival. Similar findings have been recently reported in other PLA-PEG-based drug nanocarriers, demonstrating the potential of nanomedicine for cancer treatment. In particularly, PEG has been shown to enable site-specific delivery of drugs in various cancers (Xue et al., 2018; Duncan et al., 2019; Heshmati Aghda et al., 2020; Lundsten et al., 2020). Furthermore, no significant cytotoxicity of the blank PMs was observed in ALL cells *in vitro*, even when administered at maximum doses; and we didn’t find obvious adverse reactions, such as gastrointestinal reactions, neurotoxicity, hepatoxicity and bone marrow suppression in the animals following DOX-PMs-NPMBP treatment. Compared with control or DOX alone, more mature cells were observed in the peripheral blood and bone marrow samples from DOX-PMs-NPMBP-treated animal. Herein, the *in vivo* experiments indicate that DOX-PMs-NPMBP regimen can enhance the sensitivity of ALL xenograft models to DOX and reach higher therapeutic effects with less systematic toxicity.

In conclusion, DOX-PMs-NPMBP nanoparticle can significantly inhibit leukemia cell growth and induce apoptosis; and markedly improve the antileukemia activity in resistant ALL cells *in vitro* and *in vivo*. Therefore, it is worthy of investigation in large animal models.

## Data Availability

The raw data supporting the conclusions of this article will be made available by the authors, without undue reservation. The RNA sequencing datasets presented in this study can be found in online repositories. The names of the repository/repositories and accession number(s) can be found below: https://www.ncbi.nlm.nih.gov/Traces/study/?acc=PRJNA668604&amp;o=acc_s%3Aa.
