# Supplementary figures and images for: Doxorubicin/Nucleophosmin Binding Protein-Conjugated Nanoparticle Enhances Anti-leukemia Activity in Acute Lymphoblastic Leukemia Cells in vitro and in vivo
Source: Front Pharmacol. 2021 May 28;12:607755. doi: 10.3389/fphar.2021.607755 (PMC8193937; doi:10.3389/fphar.2021.607755)

**Sup Fig.2 KEGG pathway enrichment analysis**

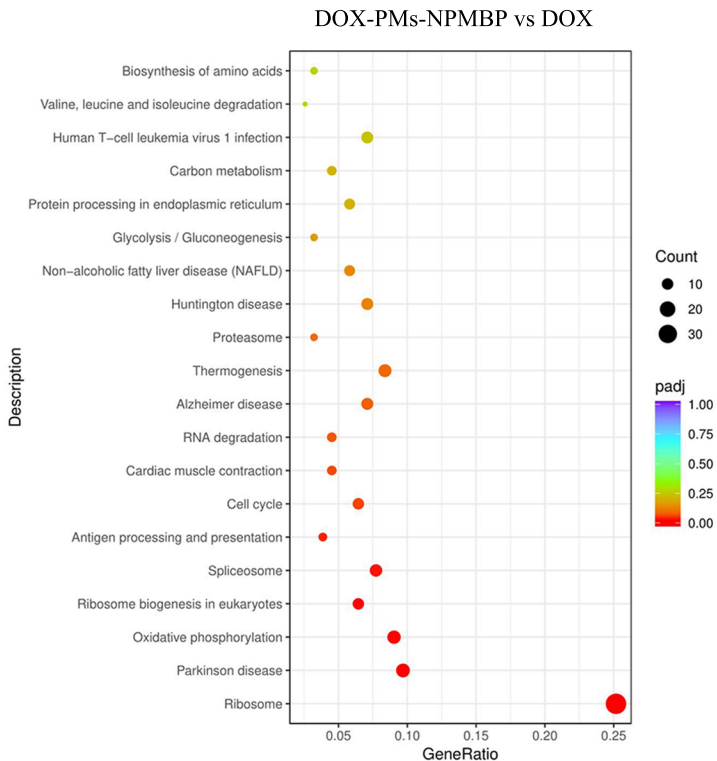

Supplement: Supplementary file 1 [file Image2.pdf]
