# Supplementary material for: Doxorubicin/Nucleophosmin Binding Protein-Conjugated Nanoparticle Enhances Anti-leukemia Activity in Acute Lymphoblastic Leukemia Cells in vitro and in vivo
Source: Front Pharmacol. 2021 May 28;12:607755. doi: 10.3389/fphar.2021.607755 (PMC8193937; doi:10.3389/fphar.2021.607755)
Supplement: Supplementary file 2 [file Image1.pdf]

Sup Fig.1

A

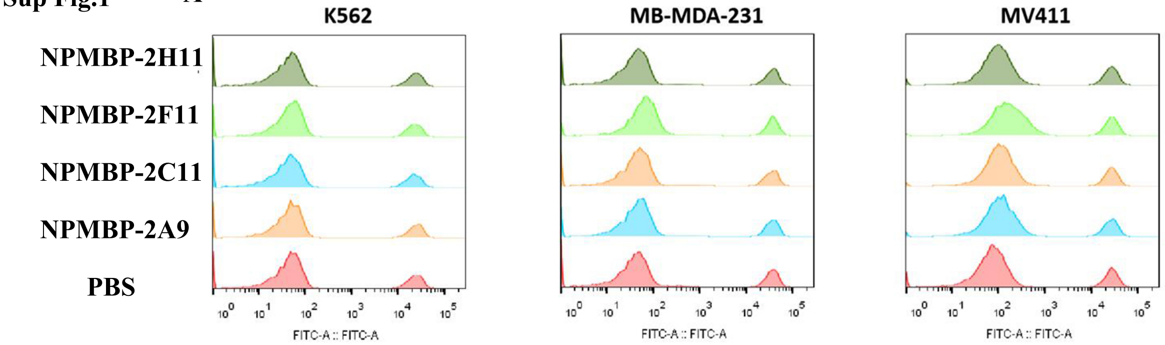

B

	MFI		
	K562	MB-MDA-231	MV411
**PBS**	52.9±5.77	50±5.86	88.7±6.33
**NPMBP-2A9**	51.6±4.38	58.7±5.77	131±7.77
**NPMBP-2C11**	59.9±2.76	63±4.35	136±6.85
**NPMBP-2F11**	54.2±4.69	82.5±3.26	209±9.88
**NPMBP-2H11**	51.9±5.88	52.3±3.37	111±3.36

K562: without NPM expression in the cell membrane

MB-MDA-231 and MV411: with NPM expression in the cell membrane
